# Epstein-Barr virus reactivation triggers selective IL-6/IL-10 axis inflammation and CD3^+^CD8^+^ T-cell activation leading to severe leukopenia, hyperinflammatory shock, and myocardial injury: a case report

**DOI:** 10.3389/fimmu.2026.1833536

**Published:** 2026-07-10

**Authors:** Yong Chen, Jie Wang, Qiuju Yao, Junhui Zhong, Jin Cao, Lei Du, Lipeng Shi, Chenghu Wang, Yi Ren

**Affiliations:** Department of Classical Chinese Medicine, Chongqing Hospital of Traditional Chinese Medicine, Chongqing, China

**Keywords:** CD3+CD8+ T cells, Epstein-Barr virus reactivation, hyperinflammatory shock, IL-6/IL-10 axis, myocardial injury, severe leukopenia

## Abstract

Reactivation of Epstein-Barr virus (EBV) can lead to life-threatening complications beyond hemophagocytic lymphohistiocytosis (HLH). We report a case of severe EBV reactivation in a 24-year-old female. She had persistent high fever, severe leukopenia, hyperinflammatory shock, and myocardial injury, but lacked typical HLH features. Laboratory tests showed elevated proportions of CD3^+^CD8^+^ T cells and increased interferon-γ (IFN-γ), interleukin-6 (IL-6), interleukin-10 (IL-10), and high-sensitivity troponin. Interleukin-2 (IL-2) and tumor necrosis factor-α (TNF-α) levels were normal. The inflammatory pathway may be involved as follows: EBV infects B cells and activates specific CD8^+^ T cells. These T cells mainly secrete IFN-γ without concurrent IL-2 or TNF-α release, resulting in moderate macrophage activation and IL-6/IL-10-related inflammation. This mechanism differs fundamentally from the uncontrolled inflammation in HLH. With glucocorticoids and ganciclovir treatment, the patient’s symptoms and laboratory markers rapidly and completely resolved. This case highlights the heterogeneity of EBV inflammatory response: clinicians should recognize its atypical manifestations, differentiate it from HLH, and provide individualized treatment based on immunophenotypic profiles.

## Introduction

Epstein-Barr virus (EBV) is a widely prevalent human herpesvirus (1). After primary infection, EBV can remain latent in B lymphocytes and reactivate under immunosuppression, stress, or other infections (2–4). Most reactivations are mild; however, some patients experience severe inflammatory responses that may cause multi-organ damage or life-threatening states. Hemophagocytic lymphohistiocytosis (HLH) is the most common severe complication of EBV reactivation. HLH features uncontrolled hyperinflammation, bone marrow hemophagocytosis, and elevated soluble CD25 (5, 6). Not all EBV reactivation cases meet HLH criteria, yet some still trigger severe inflammatory injury and organ involvement. These cases deserve careful clinical attention to avoid misdiagnosis and ensure timely intervention.

EBV reactivation can present with diverse, nonspecific clinical features. This makes it difficult to distinguish from severe bacterial infections, autoimmune diseases, or hematological malignancies (7–9). Mild cases may show only fever, fatigue, and lymphadenopathy. Severe cases can progress quickly to multiple organ dysfunction. The lack of clear biological markers causes diagnostic challenges, often leading to missed or delayed diagnoses and treatment. In young patients with normal immune function, severe and atypical EBV reactivation is rare. It usually occurs without obvious predisposing factors such as immunodeficiency or underlying disease. Unclear pathogenic mechanisms and limited data make diagnosis and management harder. These challenges highlight the need for clinical vigilance and rapid assessment in suspected cases.

This report describes a young, immunocompetent female with severe EBV reactivation, presenting with high fever, leukopenia, hyperinflammatory shock, and myocardial injury, but no predisposing factors. Rapid improvement after anti-inflammatory and antiviral therapy highlights the importance of early recognition and intervention. Prompt diagnosis and targeted therapy can be life-saving in atypical EBV presentations, even when HLH criteria are absent. This case provides clinicians with new insights for diagnosing and treating unexplained inflammatory syndromes swiftly and accurately.

## Case presentations

Informed consent was obtained from the adult participants themselves.

### Case report

A 24-year-old female was admitted to the hospital with a persistent high fever for 5 days and palpitations for 2 days. Five days before admission, she developed an acute fever with a maximum temperature of 40.2 °C and palpitations. Her temperature did not improve after oral antipyretic medication. She reported no cough, sputum production, abdominal pain, diarrhea, skin rash, or bleeding tendency. Her medical history was unremarkable, and she had no history of chronic diseases, drug allergies, or hereditary or infectious diseases in her family. No recent use of specific medications known to cause cytopenia.

At admission, her vital signs were: temperature 39.8 °C, heart rate 112 beats/min, respiratory rate 22 breaths/min, and blood pressure 85/67 mmHg. Physical examination revealed no enlarged superficial lymph nodes or tonsils. Cardiac auscultation showed a regular rhythm at 112 beats/min, with no pathological murmurs. Lungs were clear to auscultation bilaterally, with no crackles or wheezes. The abdomen was soft and non-tender, with no rebound tenderness. No hepatosplenomegaly was detected. There was no lower extremity and neurological examination was unremarkable.

On admission (Day 1), the white blood cell (WBC) count was 2.44×10^9^/L. It dropped to 0.98×10^9^/L on Day 2. Plasma EBV DNA testing using plasma revealed a high viral load of 5.26×10^5^ copies/mL, and serological findings showed positive VCA−IgG, EBNA−IgG, EA−IgG, and negative VCA−IgM. consistent with recent primary reactivation. Inflammatory markers were elevated: ferritin (1593 ng/ml), C-reactive protein (CRP, 75.29 mg/L), serum amyloid A (SAA, 197.4 mg/L), and erythrocyte sedimentation rate (ESR, 52 mm/h). Cytokine analysis showed increased interleukin-6 (IL-6, 26.2 pg/ml), interleukin-10 (IL-10, 19.9 pg/ml), and interferon-gamma (IFN-γ, 21.8 pg/ml). Levels of soluble CD25 (3812 pg/ml), tumor necrosis factor-alpha (TNF-α), and interleukin-2 (IL-2) were normal. Flow cytometry revealed a high proportion of CD3^+^CD8^+^ T cells (49.18%). Myocardial injury markers were also elevated: high-sensitivity troponin I (hs-cTnI, 1.13 ng/mL), creatine kinase-MB (CK-MB, 34.58 ng/mL), and myoglobin (199.5 ng/mL). NT-proBNP(97 ng/L),Complement (C3, C4), and immunoglobulins (IgG, IgA, IgM) were normal. Negative blood culture. Anti-neutrophil cytoplasmic antibodies (ANCA), including anti-proteinase 3 and anti-myeloperoxidase, were negative. Natural killer (NK) cell activity was 15.59% (normal 15.11–23.70%). Bone marrow aspiration and biopsy showed normocellular marrow without hemophagocytosis or abnormal cells. Blood and urine cultures were negative. Chest CT showed no significant infectious lesions. Abdominal ultrasound showed the spleen to be normal in size. The ECG displayed sinus tachycardia and ST depression in leads II, III, and aVF. Echocardiography revealed a left ventricular ejection fraction of 70%, normal cardiac structure and wall motion, with no segmental wall motion abnormalities, apical ballooning, or signs of myocardial edema. Cardiac magnetic resonance imaging was not performed. Repeated electrocardiography showed no ischemic ST−T changes, arrhythmias, or dynamic evolutionary patterns indicative of myocardial injury.

After consultation with the cardiology department, there was no evidence to support the diagnoses of myocarditis, stress-induced ischemia, stress-induced cardiomyopathy, or inflammatory myocardial involvement. Based on clinical and laboratory findings, the patient was diagnosed on day 2 with Epstein–Barr virus reactivation accompanied by severe leukopenia, hyperinflammatory shock, and myocardial injury. The patient received glucocorticoid therapy with the following regimen: intravenous dexamethasone 10 mg once daily for 7 days, followed by intravenous dexamethasone 5 mg once daily for another 7 days, and then switched to oral methylprednisolone 14 mg once daily, with the dose halved every two weeks until discontinuation. Concurrently, the patient received intravenous ganciclovir 5 mg twice daily for 7 days, intravenous moxifloxacin 0.4 g once daily for 3 days, oral esomeprazole enteric-coated capsules 20 mg once daily for 42 days, and oral calcium carbonate with vitamin D3 tablets 600 mg twice daily for 42 days. Neither fluid resuscitation nor vasoactive agents were used.

After treatment, her temperature, palpitations, and blood pressure normalized rapidly. Two days after therapy began, the WBC count recovered to 3.4×10^9^/L. Inflammatory cytokines and myocardial markers dropped significantly. After Day 7, the EBV DNA load decreased to 3.2×10² copies/mL. After 16 days, all laboratory parameters normalized, and the patient was discharged in good condition. She was followed up weekly with blood tests, and glucocorticoids were tapered and discontinued. During 6 months of follow-up, the patient remained afebrile with no palpitations.

**Figure 1:**
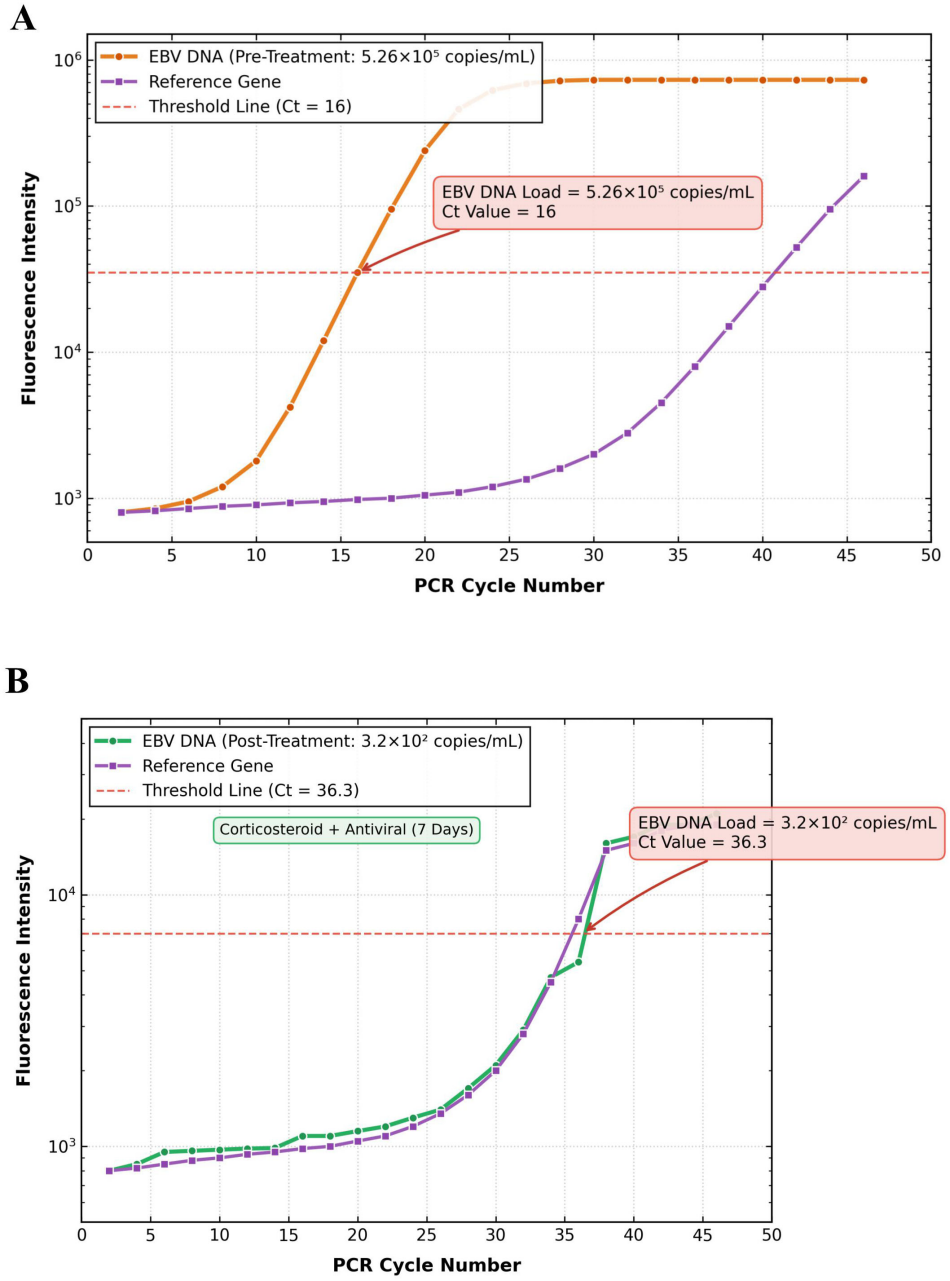
Real-time quantitative PCR amplification curves of EBV DNA targeting the BamHI-W region in plasma specimens before and after treatment in a patient with severe EBV reactivation. **(A, B)** representing pre-treatment and 7 days post-treatment, respectively. **(A)** Pre-treatment (day 1): orange solid line indicates EBV DNA (5.26×10^5^ copies/mL), purple solid line denotes the reference gene, and red dashed line shows the threshold (fluorescence intensity = 35000, Ct = 16). A typical S-shaped curve confirmed high viral load. **(B)** Seven days after glucocorticoid plus ganciclovir: green solid line indicates EBV DNA (3.2×10² copies/mL), purple solid line denotes the reference gene, and red dashed line shows the threshold (fluorescence intensity = 7000, Ct = 36.3). The post-treatment curve showed a delayed exponential phase, lower plateau fluorescence, and a higher Ct value, indicating markedly reduced EBV load. The reference gene (RNase P) shows normal amplification in both panels, confirming adequate nucleic acid extraction and the absence of PCR inhibitors.

**Figure 2:**
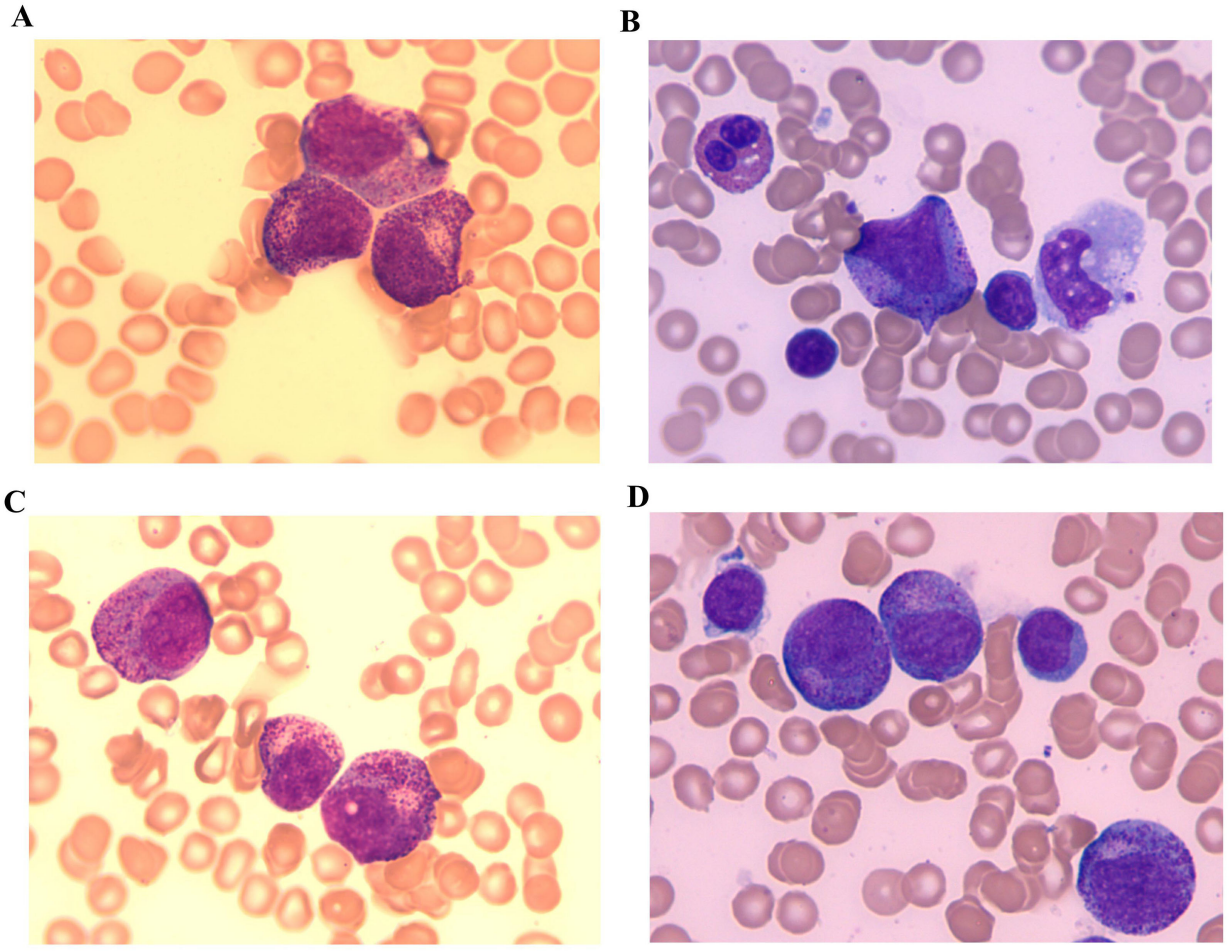
Bone marrow smear findings in a patient with severe EBV reactivation. Bone marrow smears (Wright−Giemsa staining, original magnification ×1000) from representative fields show active hematopoiesis without hemophagocytic phenomenon or significant abnormal cells. **(A)** Clusters of myeloid cells with abundant fuchsia cytoplasmic granules (consistent with activated myeloid precursors). **(B)** A mixed cellular population including segmented neutrophils, monocytes, and small lymphocytes, confirming active bone marrow hyperplasia. **(C)** Scattered myeloid cells with prominent cytoplasmic granulation, further supporting generalized myeloid activation. **(D)** Early myeloid precursors with fine nuclear chromatin and normal morphology (no atypia or malignant features). These findings collectively confirm active bone marrow hyperplasia and exclude severe complications (hemophagocytosis, abnormal cells).

**Figure 3:**
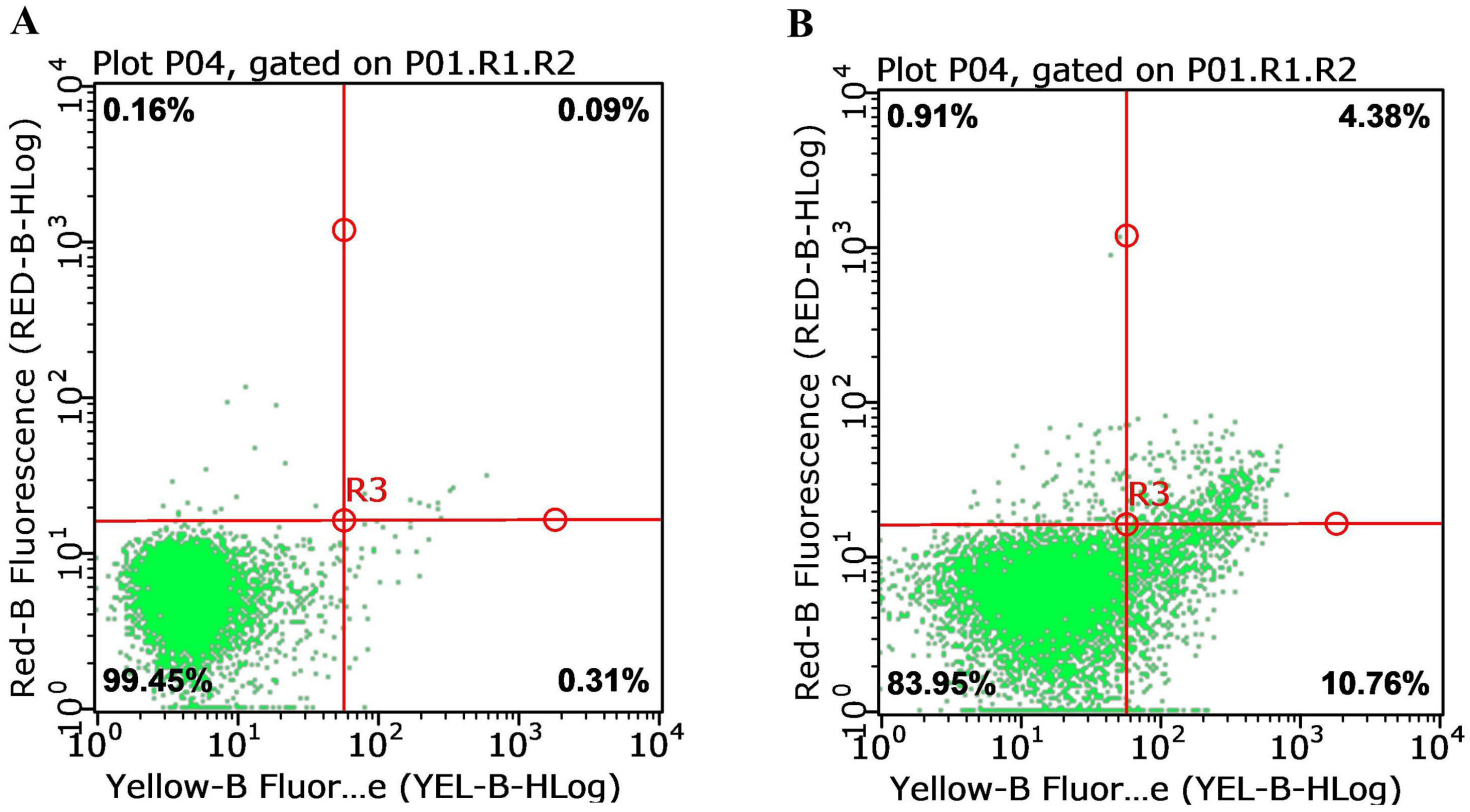
NK cell-mediated cytotoxicity against target cells in a patient with severe EBV reactivation. NK cell cytotoxicity was assessed by co−culturing patient peripheral blood mononuclear cells (PBMCs, as effector source) with Yellow−B/Red−B double−stained K562 target cells at an effector−to−target (E:T) ratio of 10:1 for 4 h at 37 °C. Spontaneous target cell death was measured in target cells alone (background control). Viable target cells were gated as R3 (P01.R1.R2) on a logarithmic scale.**(A)** Background control: Target cells without NK cells showed 99.45% viability, with 0.57% total non−viable cells (spontaneous apoptosis).**(B)** Patient sample: After co−culture with patient effector cells, viable target cells decreased to 83.95%, corresponding to 16.05% total non−viable cells. Specific lysis (15.59%, i.e., 16.05% – 0.57%) was within the normal reference range of 15.11%–23.70% (established from healthy controls), indicating preserved NK cell cytotoxic function.

**Figure 4:**
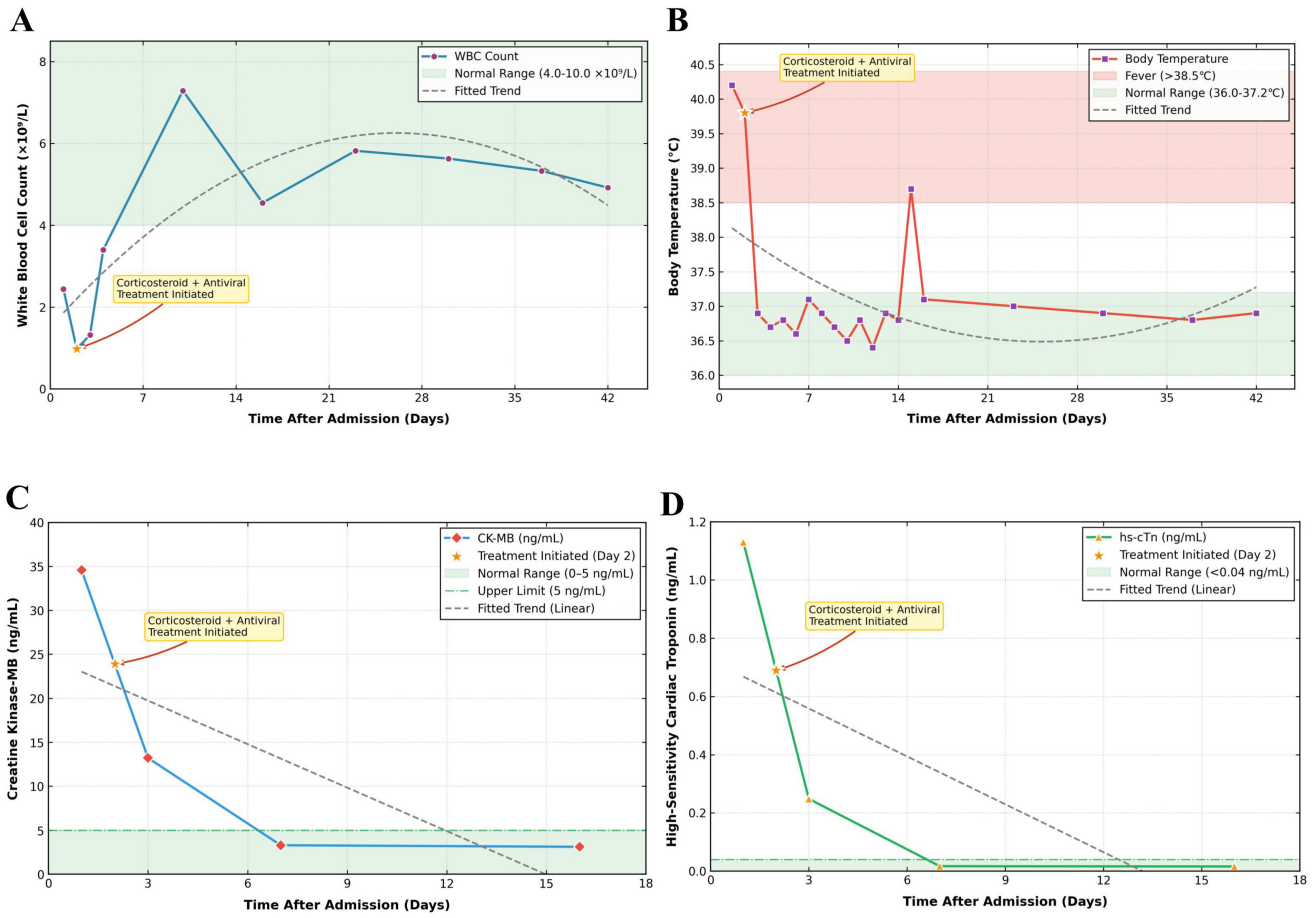
Dynamic changes of body temperature and laboratory indexes before and after treatment in a patient with severe EBV reactivation. **(A-D)** show white blood cell count, body temperature, creatine kinase-MB, and high-sensitivity cardiac troponin, respectively. **(A)** White blood cell (WBC) count was measured from day 1 to day 42; treatment was started on day 2 (orange star). The green shaded area indicates the normal range (4.0–10.0 ×10^9^/L). WBC fell to 0.98 ×10^9^/L on day 2 and recovered to normal by day 10. **(B)** Body temperature was monitored daily; the patient had a high fever (40.2°C) on day 1 and rapidly became afebrile after day 3. **(C)** Creatine kinase-MB (CK-MB) was increased (34.58 ng/mL) on day 1 and returned to normal by day 7.**(D)** High-sensitivity cardiac troponin (hs-cTn) was elevated (1.13 ng/mL) on day 1 and normalized by day 7. Green shaded areas represent normal reference ranges, and dashed gray lines indicate fitting curves.

Key changes in laboratory indices during admission and follow-up appear (**Table 1**). The timeline of pharmacological interventions (**Table 2**). Diagnostic figures include the plasma EBV DNA PCR amplification curve (**Figure 1**), bone marrow smear showing normocellularity and no hemophagocytosis (**Figure 2**), and preserved NK cell function (**Figure 3**). Rapid defervescence occurred after treatment. Peripheral blood counts and myocardial injury markers gradually normalized (**Figure 4**).

**Table 1:** Dynamic changes of laboratory during hospitalization and post-discharge.

Variables	Reference range	Day 1	Day 2	Day 3	Day 4	Day 5	Day 7	Day 8	Day 10	Day 16	Day 2 Post-disch.	Day 9 Post-disch.	Day 16 Post-disch.	Day 23 Post-disch.
Hematology Panel [WBC/Neut#/Eos#/Bas#/Mono#/Lymph#/Hb (g/L)/PLT] (×10^9^/L)	4.0–10.0/2.0–7.5/0.02–0.52/0.00–0.10/0.20–1.00/0.80–4.00/120–160/100–300	2.44/1.81/0.01/0.00/0.25/0.37/134/136	0.98/0.30/0.00/0.01/0.14/0.53/102/103	1.32/0.28/0.01/0.00/0.41/0.62/106/110	3.40/1.04/0.02/0.01/0.26/1.07/105/130	–	–	–	7.29/4.92/0.01/0.01/0.45/1.90/115/115	4.55/2.73/0.00/0.01/0.35/1.79/111/202	5.82/3.10/0.14/0.05/0.25/2.28/134/182	5.63/2.34/0.20/0.04/0.32/2.73/133/247	5.33/3.58/0.25/0.01/0.45/1.04/139/191	4.92/3.20/0.06/0.00/0.24/1.42/139/218
Cytokine[IL-6/IL-10/TNF-α/IL-2/IFN-γ(pg/mL)]	0–7/0–9.5/0–8.5/0-12/0-10	26.2/19.9/1.88/2.3/21.8	–	–	3.1/1.7/1.31/2.7/3.9	–	–	–	2.7/1.9/1.11/1.73/2.18	–	–	–	–	–
Inflammatory Markers [Ferritin/PCT (ng/mL); sCD25 (pg/mL); ESR (mm/h);CRP/SAA(mg/L)]	30–400/<0.05/0-6000/0-5/0–15/0-10	–/0.15/–/52/75.29/197.43	1953/0.93/–/–/48.18/>320	–	605.3/0.087/–/–/26.97/129.95	–/–/3812/–/–/–	286.5/0.93/–/39/–/–	–	49.9/–/–/–/8.22/–	–	–	–	–	–
Arterial Blood Gas [PaO_2_/FiO_2_ Ratio; Lac (mmol/L)]	300–500/0.5–2.2	443/1.4	455/2.8	470/1.2	–	–	–	–	–	–	–	–	–	–
Viral Serology [VCA-IgM/VCA-IgG/EA-IgG/EBNA-IgG/ EBV-DNA(copies/mL)]	Negative/Negative/Negative/Negative/**<**500	–	–/+/+/+/5.26×10^5^	–	–	–	–	–/–/–/–3.2×10²	–	–	–	–	–	–
Immune Profile [C3/C4/IgG/IgM/IgA(g/L);ANA/ENA;PR3-ANCA/MPO-ANCA(RU/mL);CD3+CD8+%]	0.7–1.4/0.1–0.4/8.6–17.4/0.5-2.8/0.72-4.29/Negative/Negative/0-20/0-20/8.99-36.72	–	–/–/–/–/–/–/–/–/–/49.18	1.06/0.24/16.36/0.60/2.93/–/–/13.46/<2/–	–	–	–	–	–	–	–	–	–	–
Myocardial injury markers[hs-cTnI/CK-MB/Myo (ng/mL)]	0–0.04/0–5/0–110	1.13/34.58/199.5	–	–	0.248/13.21/145.21	–	0.017/3.29/43.78	–	–	0.016/3.11/35.21	–	–	–	–
NT-proBNP(ng/L)	0-114.5	–	97	–	–	–	–	–	–	–	–	–	–	–
Blood culture	Negative	–	–	–	–	–	–	–	–	–	–	–	–	–
Metabolic/Coagulation[TG(mmol/L)/FIB(g/L)]	0.4-1.7/2-4	1.33/3.74	–	–	–	–	–	–	–	–	–	–	–	–

WBC, White Blood Cell Count; Neut#, Neutrophil Count; Eos#, Eosinophil Count; Bas#, Basophil Count; Mono#, Monocyte Count; Lymph#, Lymphocyte Count; Hb, Hemoglobin; PLT, Platelet; IL−6, Interleukin−6; IL−10, Interleukin−10; TNF−α, Tumor necrosis factor−α; IL−2, Interleukin−2; IFN−γ, Interferon−γ; PCT, Procalcitonin; sCD25, Soluble Interleukin-2 Receptor; CRP, C-Reactive Protein; ESR, Erythrocyte Sedimentation Rate; SAA, Serum amyloid A; PaO_2_/FiO_2_, Arterial Oxygen Partial Pressure/Fraction of Inspired Oxygen; Lac, Lactic Acid; VCA-IgM, Viral Capsid Antigen IgM; VCA-IgG, Viral capsid antigen IgG; EA-IgG, Early antigen IgG; EBNA-IgG, Epstein-Barr virus nuclear antigen IgG; EBV-DNA, Epstein-Barr virus DNA; C3, Complement 3; C4, Complement 4; IgG, Immunoglobulin G; IgM, Immunoglobulin M; IgA, Immunoglobulin A; ANA, Anti-nuclear antibody; ENA, Extractable nuclear antigen; PR3-ANCA, Proteinase 3 anti-neutrophil cytoplasmic antibody; MPO-ANCA, Myeloperoxidase anti-neutrophil cytoplasmic antibody; hs-cTnI, high-sensitivity cardiac troponin I; CK-MB, Creatine kinase-MB; Myo, Myoglobin; TG, Triglycerides; FIB, Fibrinogen; Post-disch., Post-Discharge. "–" indicates the test was not performed or the result was unavailable;"+"indicates the test was positive. Reference ranges are based on the Clinical and Laboratory Standards Institute (CLSI) guidelines for adults.

**Table 2:** Timeline of pharmacological treatment during hospitalization and post-discharge.

Drug Name	Day 1	Day 2	Day 8	Day 16	Day 2 Post-disch.	Day 9 Post-disch.	Day 16 Post-disch.	Day 23 Post-disch.
MXFX Inj.	0.4g,qd,IV,3days	–	–	–	–	–	–	–
Ganciclovir Inj.	–	5mg,q12h,IV,7days	–	–	–	–	–	–
Dex Inj.	–	10mg,qd,IV,7days	–	–	–	–	–	–
Esomeprazole EC Caps.	–	20mg,qd,PO,42days	–	–	–	–	–	–
Calcium Carbonate + Vit D3 Tabs.	–	600mg,bid,PO,42days	–	–	–	–	–	–
Dex Inj.	–	–	5mg,qd,IV,7days	–	–	–	–	–
MP Tabs.	–	–	–	14mg,qd,PO,7days	–	–	–	–
MP Tabs.	–	–	–	–	7mg,qd,PO,7days	–	–	–
MP Tabs.	–	–	–	–	–	3.5mg,qd,PO,7days	–	–
MP Tabs.	–	–	–	–	–	–	1.75mg,qd,PO,7days	–
MP Tabs.	–	–	–	–	–	–	–	0.875mg,qd,PO,7days

IV, Intravenous; PO, Per os (Oral); qd, Quaque die (Once daily); q8h, Quaque 8 hora (Every 8 hours); q12h, Quaque 12 hora (Every 12 hours); Post-disch., Post-discharge; MXFX Inj., Moxifloxacin Injection; Ganciclovir Inj., Ganciclovir Injection; Dex Inj., Dexamethasone Injection; Esomeprazole EC Caps., Esomeprazole Enteric-Coated Capsules; Calcium Carbonate + Vit D3 Tabs., Calcium Carbonate and Vitamin D3 Tablets; MP Tabs., Methylprednisolone Tablets. "–" indicates the test was not performed or the result was unavailable.

## Discussion

Hemophagocytic lymphohistiocytosis (HLH) is the most common severe complication of EBV reactivation, characterized by EBV-induced dysfunction of natural killer (NK) cells and CD8^+^ T cells, which subsequently triggers uncontrolled hyperinflammatory responses, massive cytokine release, bone marrow hemophagocytosis, and significantly elevated soluble CD25 (sCD25). In contrast, the patient met the following three of the eight diagnostic criteria for HLH (10): fever (body temperature >38.5 °C for >7 days), elevated ferritin (serum ferritin >500 μg/L), and cytopenia (neutrophils <1.0 × 10^9/L). However, spleen size was normal; hemoglobin was >90 g/L; platelet count was >100 × 10^9/L; soluble interleukin-2 receptor (sCD25) level was normal; natural killer (NK) cell activity was normal; fasting triglycerides were <3.0 mmol/L; fibrinogen was >1.5 g/L; and bone marrow smear showed no evidence of hemophagocytosis. This finding shows that EBV reactivation can cause severe organ damage via non-HLH pathways, supporting the recent view that severe inflammatory responses associated with EBV reactivation are not limited to the HLH subtype (11). This case expands our understanding of the clinical heterogeneity of EBV reactivation.

The clinical manifestations of the patient in this case were easily confused with severe bacterial infections, autoimmune diseases, and hematological malignancies, which is a common misunderstanding in the diagnosis and treatment of EBV reactivation in clinical practice (12, 13). The patient had no clear predisposing factors for EBV reactivation (such as immunosuppression, chronic underlying diseases, severe stress, etc.), and initial laboratory examinations indicated severe leukopenia and significantly elevated inflammatory markers, which were highly similar to the clinical characteristics of severe bacterial infections. However, blood and urine cultures were negative, ruling out the possibility of bacterial infection; complement C3, C4, immunoglobulins (IgG, IgA, IgM), and autoantibodies (anti-proteinase 3 antibody, anti-myeloperoxidase antibody) were all normal, excluding autoimmune diseases; bone marrow aspiration and biopsy showed no abnormal cells or hemophagocytosis, further ruling out hematological malignancies and HLH. Finally, the significantly elevated EBV DNA load (5.26×10^5^ copies/mL) and the characteristic serological pattern (positive VCA−IgG, EBNA−IgG, and EA−IgG, and negative VCA−IgM) provided the key basis for confirming recent EBV reactivation and served as an important reference for clinical differential diagnosis.

EBV reactivation and B-cell infection were the initial triggering links in the pathological process in this case (14, 15). EBV mainly completes infection by binding to CD21 molecules on the surface of B lymphocytes. After viral reactivation, it specifically expresses viral antigens such as EBNA and VCA. These antigens are recognized by the host immune system, initiating a specific cell-mediated immune response (11, 16). In this case, the significantly elevated EBV DNA load confirmed active viral replication, while the serological pattern (positive VCA−IgG, EBNA−IgG, EA−IgG, and negative VCA−IgM) indicated recent viral reactivation rather than primary infection. This suggests that the host immune response was mainly directed against viral antigens expressed after reactivation, rather than newly infected B cells, laying the foundation for the specific activation of subsequent immune responses.

Selective activation of CD3^+^CD8^+^ T cells was the core link in the pathogenesis of this case (17–19). Flow cytometry showed a significantly elevated proportion of CD3^+^CD8^+^ T cells in the patient (49.18%; normal range 8.99%~36.72%), indicating a marked expansion of the CD3^+^CD8^+^ T cell population, which includes cytotoxic T lymphocytes (CTLs). CTLs play a key role in clearing EBV-infected cells by recognizing viral antigens presented on the surface of infected B cells (20). In the pathological process of HLH, EBV-induced CTL dysfunction leads to their uncontrolled proliferation and massive release of cytokines such as IL-2, TNF-α, and IFN-γ, ultimately triggering a fatal cytokine storm (21). In contrast, CTL activation in this case showed clear selective characteristics: only IFN-γ secretion was significantly increased (21.8 pg/ml), while IL-2 and TNF-α levels remained normal. This selective cytokine secretion pattern suggests that CTL activation was in a regulatory rather than a dysfunctional state, a key difference from the pathological mechanism of HLH, indicating that the inflammatory response was controllable rather than uncontrolled.

In EBV-related inflammation, aberrant expression of IL-6 and IL-10 has been extensively reported. Previous studies have shown that both IL-6 and IL-10 levels are significantly elevated in children with active Epstein-Barr virus (EBV) infection (22). IL-6 > 20.79 pg/mL and IL-10 > 39.87 pg/mL can more accurately distinguish between EBV-associated lymphoma-associated hemophagocytic lymphohistiocytosis and non-neoplastic EBV-associated hemophagocytic lymphohistiocytosis (23). A selective inflammatory response mediated by the IL-6/IL-10 axis may contribute to organ damage in this case. IFN-γ secreted by activated CTLs can induce moderate activation of macrophages (24), and activated macrophages further secrete IL-6 and IL-10, forming a specific inflammatory axis. Among them, IL-6, a core pro-inflammatory cytokine, plays a key role in triggering fever, promoting the synthesis of acute-phase response proteins, and mediating tissue damage (25). In this case, the significantly elevated IL-6 level (26.2 pg/ml) was closely related to the patient’s severe leukopenia, hyperinflammatory shock, and myocardial injury: on the one hand, IL-6 can directly inhibit bone marrow hematopoietic function, leading to a significant decrease in white blood cell count (26); on the other hand, IL-6 can induce vascular endothelial dysfunction, increase vascular permeability, reduce vascular resistance, and participate in the occurrence and development of hyperinflammatory shock. In contrast, IL-10, an important anti-inflammatory cytokine, can prevent the onset of a cytokine storm by antagonizing excessive inflammatory responses and inhibiting the release of pro-inflammatory cytokines (27). The elevated IL-10 level (19.9 pg/ml) in this patient is speculated to be a compensatory anti-inflammatory response of the body to limit the further aggravation of inflammatory damage, which also explains why the inflammatory response in this case was controllable and did not develop into the uncontrolled hyperinflammatory state seen in HLH. Therefore, the dynamic balance between IL-6 (pro-inflammatory) and IL-10 (anti-inflammatory) may be involved in the core feature of the unique pathogenesis in this case and may serve as a key distinguishing factor from HLH.

All organ damage in the patient was closely related to the above-mentioned selective inflammatory response. Severe leukopenia (minimum 0.98×10^9^/L) is speculated to be related to two factors: first, the direct inhibitory effect of IL-6 on bone marrow hematopoietic function; second, the direct cytotoxic effect of activated CD3^+^CD8^+^ T cells on bone marrow hematopoietic cells (28). The occurrence of hyperinflammatory shock was mainly due to IL-6-induced vascular endothelial dysfunction, increased vascular permeability, and decreased vascular resistance, leading to circulatory disorders (29). There was clear evidence of myocardial injury, with significantly elevated levels of high-There was clear evidence of myocardial injury, with significantly elevated levels of high-sensitivity troponin I, CK-MB, and myoglobin. Its occurrence may occur through two pathways: first, direct invasion of cardiomyocytes by EBV; second, indirect myocardial injury caused by IL-6-mediated inflammatory response (30). The ST-segment depression in leads II, III, and aVF on the electrocardiogram further confirmed myocardial ischemia due to inflammatory damage.

The treatment regimen of glucocorticoids (dexamethasone) combined with ganciclovir ultimately achieved complete remission of the patient’s clinical symptoms and laboratory indicators. Among them, ganciclovir inhibits viral replication by targeting EBV DNA polymerase, reduces viral antigen production, and thereby inhibits the activation of CD3^+^CD8^+^ T cells at the source (31); glucocorticoids mainly inhibit macrophage activation and IL-6 secretion, reducing organ damage caused by inflammatory responses (32). After treatment, the patient’s body temperature returned to normal quickly, the white blood cell count recovered, and inflammatory markers, myocardial injury markers, and EBV DNA load gradually decreased to normal levels. This may be explained by the selective activation of CD3^+^CD8^+^ T cells and the inflammatory response mediated by the IL-6/IL-10 axis, which are key pathogenic factors in this case, and also verifies the effectiveness of the combined treatment regimen targeting viral replication and the inflammatory response in such cases.

This study has certain limitations that need to be improved in subsequent studies: first, this study is a single case report with a limited sample size, and the generalizability of the research conclusions needs to be further verified in a larger sample cohort study; second, the expression of EBV viral antigens (such as EBNA1, LMP1) in B cells and CD3^+^CD8^+^ T cells was not detected, which cannot further intuitively confirm the interaction between EBV-infected B cells and CTLs, making it difficult to more accurately explain the triggering mechanism of immune activation; third, dynamic monitoring of T cell subsets and cytokines was not performed during treatment, so the evolution process of the immune response and inflammatory axis cannot be clearly presented; fourth, the proposed mechanism of selective activation of CD3^+^CD8^+^ T cells and IL-6/IL-10 axis-mediated inflammation still needs to be further verified by basic experimental studies to clarify its molecular regulatory pathway; fifth, given the single-case nature of this study and the lack of mechanistic assays, these findings should be interpreted as hypothesis-generating rather than definitive evidence, as the proposed mechanism is only a research hypothesis based on clinical observations, literature reports, and laboratory-associated indicators, rather than a proven causal pathway; Sixth, the patient had normal blood routine on day 23 post-discharge and reported no fever, palpitations, or adverse reactions to glucocorticoid therapy during 6 months of follow-up; however, no follow-up assessments were performed for blood routine, EBV DNA, inflammatory markers, cardiac biomarkers, or electrocardiography.

In conclusion, this case clarifies the unique pathogenesis of non-HLH severe EBV reactivation, which differs from the uncontrolled inflammatory cascade of HLH. Combining the patient’s laboratory results, treatment response, and relevant literature, we propose that EBV reactivation may be involved in potential pathogenic pathway: EBV infection of B cells, followed by selective activation of CD3^+^CD8^+^ T cells. These activated T cells exhibit a specific pattern of IFN-γ secretion, without concomitant IL-2 or TNF-α release. This cascade subsequently leads to moderate activation of macrophages and initiation of an IL-6/IL-10 axis−mediated controllable inflammatory response, ultimately culminating in severe organ damage, including severe leukopenia, hyperinflammatory shock, and myocardial injury. Treatment with glucocorticoids combined with ganciclovir can effectively inhibit viral replication and the inflammatory response, achieving complete remission in the patient and confirming the effectiveness of this treatment regimen for such non-HLH severe EBV reactivation cases. This case further enriches the clinical and pathological characteristics of non-HLH severe EBV reactivation, clarifies its unique pathogenesis, and provides an important clinical reference for the differential diagnosis, precise treatment, and prognosis evaluation of such cases in clinical practice.

## Data Availability

The original contributions presented in the study are included in the article/supplementary material. Further inquiries can be directed to the corresponding authors.
